# Variable bin size selection for periestimulus time histograms (PSTH) with minimum mean square error criteria

**DOI:** 10.1186/1471-2202-16-S1-P80

**Published:** 2015-12-18

**Authors:** SM Heidarieh, M Jahed, A Ghazizadeh

**Affiliations:** 1 Electrical Engineering, Sharif University of Technology, Tehran, Iran; 2 Laboratory of Sensorimotor Research, National Institutes of Health, Bethesda, MD, USA

## 

To date the most common method for extracting neuronal responses is by constructing PSTHs that are time locked to task events. Many parameters of interests such as response magnitude, onset and duration are then calculated from the constructed PSTHs. However the precision of PSTH response estimate critically depends on the choice of bin sizes. This dependence demands objective criteria for bin size selection. A seminal study by Shimazaki and Shinomoto [1] derived an optimal cost function for choosing a fixed bin size for a time varying Poisson process. It is easy to see that using a one-size-fit-all recipe for bin sizes will invariably overestimate and underestimate rate changes for fast and slow fluctuations respectively.

**Figure 1:**
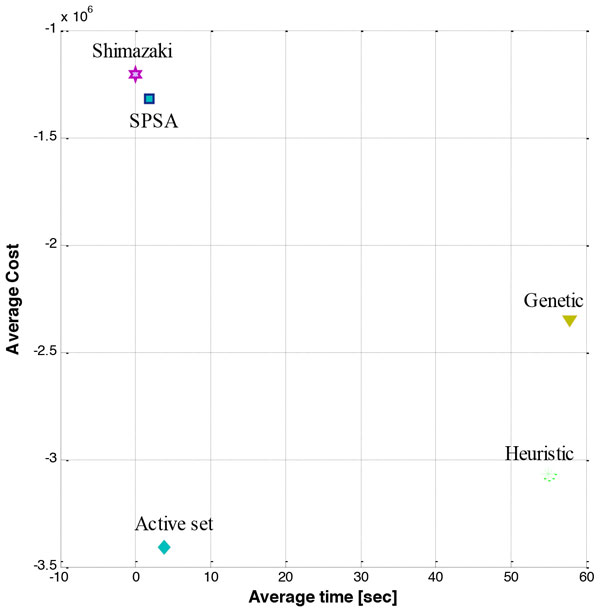
**Comparing the costs and time efforts of the algorithms**.

Here we extend previous results by calculating the cost function that minimizes mean square error for variable bin sizes with the same assumptions used previously for time varying Poisson processes $Cost\left(N,\vec{\Delta }\right)=\frac{1}{n^{2}T}\sum_{i=1}^{N}\left(\frac{2k_{i}-{\left(k_{i}-\Delta_{i}\bar{k}\right)}^{2}}{\Delta_{i}}\right)$. To minimize this nonlinear and nonconvex cost function, we utilize an array of methods some of which are widely used for nonlinear optimization, namely: Active set, Simultaneous perturbation stochastic approximation (SPSA), Genetic Algorithm and an approximate heuristic algorithm. Average performance of each algorithm on a typical simulated neuronal firing is calculated using 50 iterations. All methods resulted in a lower cost function compared to fixed bin size as expected. Plotting the final cost vs the algorithm run time shows that the method of 'Active set' overall has the best cost reduction while still being reasonably fast compared to the fixed bin size approach (Figure 1) . Further investigation of the properties of this cost function and developing computationally efficient methods for its minimization will be the basis of future work.
